# Clinical Lecture upon Hare-Lip, Cleft Palate and Epithelioma

**Published:** 1893-02

**Authors:** C. G. Darling


### Clinical Lecture Upon Hare-Lip, Cleft Palate, and Epithelioma.

BY C. G. DARLING, M.D.,
Demonstrator of Surgery in the Medical Department of the University of Michigan, and Clinical Lecturer on Oral Pathology and Surgery in the College of Dental Surgery.

Ladies and Gentlemen:—The first case which I present to you to-day, is one of common deformity—hare-lip. The extensive complications which accompany this case are, however, exceedingly rare.

When I turn this babe toward you, two deep fissures may be seen extending from the border of the lip to the nose, and leave a prominent part, the inter-maxillary portion, projecting forward from the end of the nose. When the mouth is open you will see that the cleft on the left side extends entirely through the hard and soft parts of the palate, exposing the roof of the nares and nasal portion of the pharynx.

The child is eight months old, apparently enjoying good health, and appears well nourished.

This is exceptional, for children with this deformity do not come up to the usual standard of good health. First, they do not seem to have a strong constitution by inheritance, hence few of them reach manhood and womanhood, as may be seen by comparison of the number of adults who show the marks of operation with the number of children who are presented for operation. Second, these children cannot nurse, because they cannot hold a nipple in the mouth without closing the nares, thus making it impossible to breathe. They must be fed with a spoon, a slow and laborious task for mother or nurse, and we may well doubt if the appetite is at all times satisfied. It would require a number of lectures on the subject of embryology to fully explain to you the cause of this deformity, which is really an arrested development of the child in utero.

You may have noticed that cases presented here have hare lip without cleft palate, and cleft palate without hare-lip, such as the case presented at the last clinic. Now, these conditions will depend largely on the time when development is arrested, and other conditions not fully understood.

You will observe that the cleft in the palate is in the median line until it reaches the alveolar border, then deviates toward the left and ends in the left cleft of the lip. Thus you see cleft palate in the median line—hare-lip never—if there is regular development up to the time of their formation.

The process is briefly as follows: Early in the formation of the fœtus, the buccal cavity appears as a wide cleft with no outline of the face. The fronto-nasal process, which goes to form the nose, and the inter-maxillary process, are seen above; and the superior maxillary processes on either side. At this stage of development the nasal and buccal cavities are one. Soon the pterygo-palatine processes begin to develop from the maxillary processes and advance toward the median line. The inter-maxillary process now comes down to fill the space between the superior maxillary processes and complete the alveolar arch. When development continues in the usual manner, the pterygo-palatine processes fuse in the median line, and the mouth and face are completely formed.

The process of fusing begins in the anterior portion about the eighth week, and is completed in the soft palate about the ninth or tenth week of fœtal life. If the process of fusion is arrested in an early stage, before the inter-maxillary process comes down, a double cleft in the lip may result as we see it in the child, or the inter-maxillary process may come down, uniting to the superior maxillary process on one side and the cleft remaining on the opposite side, forming single hare-lip. When the pterygo-palatine plates fail to meet at any point in the median line, a cleft remains, involving the soft and hard palate or, if the process be more nearly complete before development is arrested, a cleft in the soft palate plate remains as in the case presented at the last clinic.

The cause of arrested development is little understood, though much is attributed to maternal impressions and their effect upon the child during the early months of pregnancy.

Rokitansky asserts that mental impressions on the mother do influence the development of the embryo and often cause marked malformations.

Dabney (Keatings' "Cyclopedia of Children's Diseases") has given a very complete table of ninety authentic cases which he has collected; he also gives the name of the reporter, the journal where the report may be found; the time in pregnancy when the maternal impression was supposed to have been made, the nature of the impression and the defect it produced on the child.

Twenty-one of these ninety cases of deformity were hare-lip and cleft palate. The time when the impression was made and the variety of impressions are so numerous that I consider it important to mention a few of them.

A woman not many weeks pregnant saw a beggar with a hare-lip. She became impressed from that moment that her child would have the same defect, and explained her fears to her friends. Her child had a hare-lip. Another case reported was a woman in the fourth month of pregnancy who saw a child killed—the incisor teeth were driven up into the jaw. Her child was born with hare-lip.

Another was frightened by a rabbit, and the child presented the same defect in the lip.

The father of the boy presented at the last clinic believes that the defect is the result of a maternal impression, which he gave as follows: During the fourth month of pregnancy the mother nursed two sick children through a very severe sickness. She wept much and had a constant irritation of the soft palate. Her child was born with a cleft palate.

A woman worked in a factory with a man who had cleft palate. She did not see him during pregnancy, but was apprehensive lest her child should have a similar defect. Her child was born with cleft palate.

These cases suffice to show that time of receiving impressions may vary from some time previous to the pregnancy to four months after conception, the average time being two and a half months—about the time when the cleft should be closing or nearly closed.

We can readily believe that impressions received before the third month may be sufficient to account for these malformations, but those cases where the impressions came later than the fourth month of pregnancy, must cause some doubt of the theory, which our present knowledge of embryology will not fully explain. It may be possible that the recent union of these tissues as they are joined together, is dissolved, and a separation occurs.

Enough proof has been found to warrant us in saying that maternal impressions may be sufficient to cause such defects as hare-lip and cleft palate in the child, provided they occur before pregnancy or in the early months of pregnancy.

In many of these cases the defect has a striking resemblance to the impression, while in a few instances they seem to bear no direct relation, one to the other. In some cases the defect is anticipated, while in others it becomes an afterthought—the child has the defect and the mother depends on her memory to recall the impression which may have caused it. The impression is generally profound, comes suddenly and often produces great shock.

The mother of the child before us knows of no shock or impression which might account for the defect. There is nothing in the family history to suggest that it comes by inheritance, for hare-lip may come in families for generations, enough to lead us to think that it may be inherited.

Now comes the question of repair: What can the surgeon do toward completing the work which nature has left undone?

The surgeon can begin this work by repairing the lip, but it must be completed years hence by the capable dentist when he fashions proper mechanical appliances to so occupy the space left in the hard and soft palate that it shall in a measure compensate for their loss in speech.

The next case is Mr. ——, seventy-four years old, a farmer with a good family history. He is by no means an old man for his years, has been a temperate man all his life, but—chews tobacco moderately. This hard mass which you see on the lower lip, near the angle of the mouth, first came to his notice about six months ago. Then it was but a small abrasion of the mucous surface of the lip, easily irritated, though not painful, and showed no tendency to heal.

Some of the lower teeth on this side were loose, and, fearing that they had not only caused the first abrasion, but were keeping up the irritation, he had them extracted. Still the wound showed no tendency to heal. You see that the only remaining tooth on this side is affected by tartar; the gum is inflamed and the tooth is loose, conditions which probably affected those teeth which were extracted.

When we examine this ulcerated surface closely, we see a fluid constantly oozing from it; the borders are irregular and hard. The induration does not extend very deep, but gives a distinct outline to the ulcerated surface. This ulcer bleeds freely when touched, a condition characteristic of all ulcers when the granulating surface is exposed.

So much for symptoms and appearance. How may we arrive at a diagnosis?

There are three distinct conditions of disease which may be present in this locality: simple ulcer, syphilis and epithelioma.

Simple ulcer comes as the result of cold, a wound or the direct application of an irritant which destroys the mucous membrane. It is common to children and persons debilitated by disease, hence they are often present during convalescence from long-continued fevers. They last but a short time, and when protected they show a tendency to heal. However, when neglected, they may become quite large, difficult to manage, and can only be cured by excision. Simple ulcer may begin as a crack, and in weak tissues may grow deeper as well as broader. The constant ooze of serum from such an ulcer finally forms a scab, which is its own protection and favors healing.

The syphilitic ulcer, chancre or primary lesion of syphilis is peculiar from its first appearance; it begins as a slight abrasion of the epithelium, so small that it is scarcely noticed; it is painless, but soon becomes indurated at the base, and is always moist on the surface. Not many days after the appearance of the primary sore, the sub-maxillary glands will be found enlarged. There may be a history of exposure to syphilis, but the peculiar appearance, the early induration of the sub-maxillary glands will be sufficient for diagnosis.

Neither of these conditions seem to accord with the history which our patient has given us, nor do I find the sub-maxillary glands enlarged.

What are the symptoms of epithelioma of the lip?

A sore, which may have begun as a fissure, an elevation of epithelium or an abrasion. Age is an influential factor in producing it; rarely is it seen before forty-five, and is more common in persons over sixty years of age. It appears more frequently on the lower lip, and near the angle of the mouth than on any other part of the body; is more common to males than females, probably because males are more frequently exposed to the conditions which produce it.

The disease is at first confined to the surface of the skin and mucous membrane, but the deeper tissues are slowly invaded by the newly-formed cells; and because of this slow infiltration the lymphatic glands are not so early involved as in syphilis. While the progress is slow, it is very certain, not being limited to skin and mucous membrane, but gradually invades the periostium and does not hesitate to destroy the maxillary bone, until a great sacrifice of tissue is necessary to stay the progress of disease.

Pain is an early symptom, sharp and stinging like the pricking of needles; not severe at first, but gradually increasing until it becomes a prominent factor in undermining the patient's health.

You see these symptoms correspond so well with the history and appearance of our patient that it leaves no doubt that his disease is epithelioma. A discharge is constantly taking place, and to remove it our patient uses his handkerchief, which, not being surgically clean, causes much irritation to the exposed surface.

The cause of epithelioma as well as other forms of cancer is not well known, but it is understood that constant irritation of a part will greatly favor its appearance. It has been observed that smokers, particularly those who use a clay pipe, are prone to have cancer of the lip, the epithelioma appearing at that point where the pipe rests upon the lower lip.

Dr. Warren made some observations on all patients with cancer of the lip who presented themselves at the Massachusetts General Hospital for treatment and found that of 77 cases (73 males and 4 females) 44 smoked pipes, and 3 of the 4 women were smokers. This is probably the experience of nearly every surgeon. The irritant may be the short clay pipe, the paper of the vile cigarette or the irritating effect of the smoke itself. Jagged teeth and accumulations of tartar are sufficient irritants to produce the disease.

Epithelioma is a local disease which tends to spread to the surrounding tissues and in time reaches the neighboring lymphatic glands. The rapidity of growth will depend somewhat on the location and stage of development. The treatment then is obvious, to use the best-known means for the early removal of the disease. The remedial measures which have been from time to time recommended, should only be used in such places as can not be reached by the knife, because their action is not always certain and valuable time may be lost in giving them a trial. I mention the use of caustics for their removal only to condemn them. Their action cannot well be controlled and they sometimes hasten the development of cancer by their extreme irritating action instead of arresting the disease. In the early stage of the disease all suspicious-looking tissue can be removed from the lip, but the operator must cut well from the indurated border if he would not leave any diseased or infiltrated tissues behind as a starting point for the return of the growth.

